# Impact of OGT deregulation on EZH2 target genes FOXA1 and FOXC1 expression in breast cancer cells

**DOI:** 10.1371/journal.pone.0198351

**Published:** 2018-06-04

**Authors:** Ewa Forma, Paweł Jóźwiak, Piotr Ciesielski, Agnieszka Zaczek, Katarzyna Starska, Magdalena Bryś, Anna Krześlak

**Affiliations:** 1 Department of Cytobiochemistry, Faculty of Biology and Environmental Protection, University of Lodz, Pomorska, Łódź, Poland; 2 Department of Otolaryngology and Laryngological Oncology, Medical University of Lodz, Kopcińskiego, Łódź, Poland; Roswell Park Cancer Institute, UNITED STATES

## Abstract

Enhancer of zest homolog 2 (EZH2) is a histone methyltransferase which plays a crucial role in cancer progression by regulation of genes involved in cellular processes such as proliferation, invasion and self-renewal. Activity and biological function of EZH2 are regulated by posttranslational modifications. It is suggested that EZH2 stability may be regulated by O-GlcNAc transferase (OGT), which is an enzyme catalyzing the addition of GlcNAc moieties to target proteins. In this study, we determined the impact of OGT on expression of EZH2 target genes *FOXA1* and *FOXC1*, that are involved in breast cancer progression. The results of chromatin immunoprecipitation experiments showed that both EZH2 and OGT are targeted to the promoter regions of *FOXA1* and *FOXC1* and knockdown of EZH2 or OGT affects expression of studied genes in breast non-malignant (MCF10A) and cancer cells (MCF7, T47D and MDA-MB-231). The results showed that OGT silencing affects EZH2 binding to *FOXC1* promoter but the effect is cell-context dependent. Despite the slight decrease in EZH2 protein level in cells with OGT depletion, EZH2 binding to *FOXC1* was increased. Moreover, OGT binding to promoter regions of *FOXA1* and *FOXC1* was increased in cells with knockdown of EZH2. Increased expression of *FOXA1* and *FOXC1* in cells with OGT deregulation was associated with increased acetylation level of histone H3. The results suggest that OGT is involved in regulation of *FOXA1* and *FOXC1* expression but its role is not associated with regulation of EZH2 protein stability.

## Introduction

Enhancer of zest homolog 2 (EZH2) is an enzymatic component of Polycomb Represive Complex 2 (PRC2) responsible for methylation of histone H3 at lysine 27 (H3K27me) which mediates silencing of target genes [1]. Deregulation of EZH2 is frequently observed in a variety of cancers and is associated with cancer initiation, development, progression, metastasis and drug resistance [2, 3]. EZH2 promotes neoplastic transformation of breast epithelial cells. Abnormally elevated EZH2 level may be a promising biomarker for aggressive breast cancers with poor prognosis [4–6]. Multiple EZH2 target genes were identified and their repression by EZH2 was associated with cancer aggressiveness. It is suggested that the forkhead box transcription factors, FOXA1 and FOXC1 are regulated by EZH2 [7, 8]. FOXA1 is overexpressed in hormone-dependent cancers, including breast cancer [9, 10]. High expression of FOXA1 in cancers is usually associated with favorable clinical outcome. In breast cells FOXA1 is required for the expression of 50% of ER regulated genes [10]. Although the earlier studies have shown that FOXA1 can act either as a growth stimulator or as a repressor, it is suggested that the crosstalk between FOXA1 and ER promotes the expression of differentiation-associated genes rather than proliferation-associated genes [10]. Unlike FOXA1, expression of FOXC1 correlates with the basal-like breast cancer subtype and predicts poor breast cancer patients outcome [11]. Overexpression of FOXC1 causes changes in expression of genes involved in epithelial to mesenchymal transition (EMT) and increases cellular invasion, migration and proliferation [11–13].

Activity and biological function of EZH2 are regulated by post-translational modifications such as phosphorylation, sumoylation or ubiquitination [3]. EZH2 has also been shown to be regulated by O-GlcNAc transferase (OGT), which is an enzyme catalyzing the addition of the N-acetylglucosamine moieties by O-linkage to target proteins [14]. Elevated O-GlcNAcylation (hyper-O-GlcNAcylation) occurs in human malignancies including breast cancer [15, 16].

Changes in O-GlcNAcylation are associated with changes in OGT and/or O-GlcNAcase (OGA) levels. OGA is an enzyme catalyzing the hydrolytic removal of the sugar moiety from proteins. Aberrant expression of O-GlcNAc cycling enzymes seems to be a characteristic feature of human cancers [15]. High expression of OGT and low expression of OGA are observed in poorly differentiated breast tumors [17].

In this study, we investigated whether OGT may affect the expression of *FOXA1* and *FOXC1* and how it is associated with EZH2 regulation. Our results showed that both OGT and EZH2 regulate *FOXA1* and *FOXC1* expression and both proteins may affect each other’s binding to *FOXC1* promoter.

## Materials and methods

### Antibodies and reagents

All chemicals were obtained from Sigma (St. Louis, MO) except as noted. Cell culture reagents and materials were purchased from Lonza (Allandale, NJ, USA) and Corning, Inc. (Corning, NY, USA). Anti-O-linked N-acetylgucosamine (RL2) mouse monoclonal antibodies were purchased from Abcam^®^ (Cambridge, UK). The antibodies against OGT, EZH2, SUZ12, methylated H3 (H3K27me3), acetylated H3 and anti-rabbit IgG were obtained from Cell Signaling Technology^®^ (Boston MA, USA). The monoclonal anti-β-actin antibody and goat anti-mouse antibody were from Santa Cruz Biotechnology (Santa Cruz, CA, USA). Anti-acetyl-histone H3 antibody was obtained from Merck Millipore (Billerica, MA, USA). Simple CHIP^®^Enzymatic Chromatin IP Kit was obtained from Cell Signaling Technology^®^ (Boston, MA, USA).

### Cell culture and treatment

Non-tumorigenic MCF10A epithelial breast cells and MCF7, T47D, MDA-MB-231 breast cancer cell lines were obtained from the American Type Culture Collection (Manassas, VA).

MCF-7, MDA-MB-231, T47D cells were grown in Dulbecco’s modified Eagle’s medium (DMEM) supplemented with 10% fetal bovine serum (FBS). MCF10A cells were grown in DMEM F-12 medium supplemented with 0.4% bovine pituitary extract (BPE), 3 ng/ml hEGF, 5 μg/ml insulin, and 0.5 μg/ml hydrocortisone. All cells were grown 37°C in 5% CO_2_ incubator.

Knockdown experiments were performed using Silencer Select siRNA (ID: s16093 for OGT, ID: s4918 for EZH2) (Ambion^®^, Carlsbad, CA USA). Cells were seeded in a 6-well tissue culture plates at a density of 6×10^5^ cells/well. To knockdown of OGT or EZH2, for each well 30 nmol/l control siRNA or siRNA targeting OGT or EZH2 were complexed to Lipofectamine RNAiMAX (Invitrogen^TM^, ThermoFisher Scientific, Grand Island, NY, USA) following manufacturer’s specifications. Overexpression of OGT was established by transfection of TrueORF Gold Expression-validated cDNA Clone pCMV6-OGT plasmid expression vector (Origene, Rockville, MD, USA) into cells with Lipofectamine™2000 (Invitrogen^TM^, ThermoFisher Scientific, Grand Island, NY, USA). For PUGNAc experiments medium was augmented with PUGNAc resulting in final inhibitor concentration of 50 μM. For each experiments cells were plated in triplicates. Experiments were repeated three times. Cells were treated for 48 h.

### RNA isolation and RT-PCR

RNA was isolated from the cells using the ExtractMe Total RNA Kit (Blirt, Poland) according to the manufacturer's instructions. First strand cDNAs were obtained by reverse transcription of 2 μg of total RNA using High Capacity cDNA Reverse Transcription Kit (ThermoFisher Scientific, Waltham, MA USA) following the manufacturer's protocol. Real-time amplification of the cDNA was performed using TaqMan® Gene Expression Assay (ThermoFisher Scientific, Waltham, MA USA) according to the manufacturer's instructions.

The fluorogenic, FAM-labeled probes and the sequence-specific primers for OGT, OGA, EZH2, SUZ12, CDH1, FOXA1, FOXC1, SLUG, ZEB1, TWIST1 and the internal control HPRT1 were obtained as inventoried assays: Hs00269228_m1, Hs002011970_m1, Hs00544833_m1, Hs00248742_m1, Hs01023894_m1, Hs04187555_m1, Hs00559473_s1, Hs00161904_m1, Hs01566407_m1, Hs01675818 and Hs02800695_m1 (Applied Biosystems, ThermoFisher Scientific, Waltham, MA, USA). Fold differences in genes expression, normalized to HPRT1 levels were calculated using the formula 2^ΔΔCt^.

### Cytoplasmic and nuclear fraction isolation

Cells were fractionated to nuclear and cytoplasmic fractions using the ProteoJET nuclear cytoplasmic proteins separation kit (Fermentas, ThermoFisher Scientific, Waltham, MA USA) according to manufacturer’s instruction.

### Western blotting

Proteins of the cell lysates or nuclear and cytoplasmic fractions were resolved by 8% SDS-PAGE and electroblotted onto Immobilon-P transfer membranes. The blots were incubated with primary antibodies for 2 h at room temperature. After washing three times with TBST (Tris-buffered saline, 0,01% Tween 20) blots were incubated for 1 h with goat anti-mouse or anti-rabbit secondary antibodies conjugated with horse-radish peroxidase. Proteins were visualized on X‑ray film by an enhanced chemiluminescence method. For loading control, blots were reprobed with anti-β-actin antibody following a stripping protocol.

### Chromatin immunoprecipitation assay

ChIP assay was performed using Simple ChIP® Enzymatic Chromatin IP Kit (Agarose beads) (Cell Signaling Technology® Boston MA, USA) according to the manufacturer's instructions. Cells were fixed using formaldehyde and washed by ice-cold PBS solution three times. After cell lysis the chromatin was harvested and fragmented by partial digestion with micrococcal nuclease to obtain chromatin fragments of 1 to 5 nucleosomes in size. The chromatin fragments were then immunoprecipitated with specific antibodies (Ab) against EZH2, OGT, trimethylated histone H3 lysine 27 (H3K27Me3) or acetylated histone H3. A control immunoprecipitation using IgG was set up in parallel to distinguish non-specific precipitation. Differential chromatin enrichment was quantified using real-time quantitative PCR. The primer pairs that covered the *FOXA1* and *FOXC1* promoter regions were: for *FOXA1* forward 5’-GTAGGTGCGAGCGTCTTTG-3’ and reverse 5’-CTTACGTCGCTTGAGTGCC-3’; for *FOXC1* forward 5’-TGTCCTTTGTAGCCGAGTTCAG-3’ and reverse 5’-GCTTTTCTCCACTTGGAGGTTC-3’. Results represent the mean of three independent experiments performed in triplicate.

### Migration/invasion assay

The transwell assay was performed to assess the rate of migration or invasion of MCF10A cells with OGT reduced expression or OGT overexpression. Assays were performed using Millicell^TM^ hanging cell culture inserts (polyethylene terephthalate PET membranes with 8 μm pores) (Merck Millipore, Billerica, Massachusetts, USA). Twenty-four hours after transfection cells (5x10^4^) were plated in serum- free medium and placed in the upper chamber, while the lower chamber was filled with serum-containing medium. Cells were cultured for 24 h. After incubation cells in upper chamber were removed and the migrated cells at the bottom of the PET were fixed in 4% paraformaldehyde and stained with Giemsa. For the invasion assay chambers were coated with Matrigel^®^ Matrix Basement Membrane (Corning, USA).

### Statistical analysis

Statistical analyzes were performed with STATISTICA 10 (StatSoft, Poland) using a t-test.

## Results

### Expression of EZH2, OGT, FOXA1 and FOXC1 in breast cancer cells

**Fig 1:**
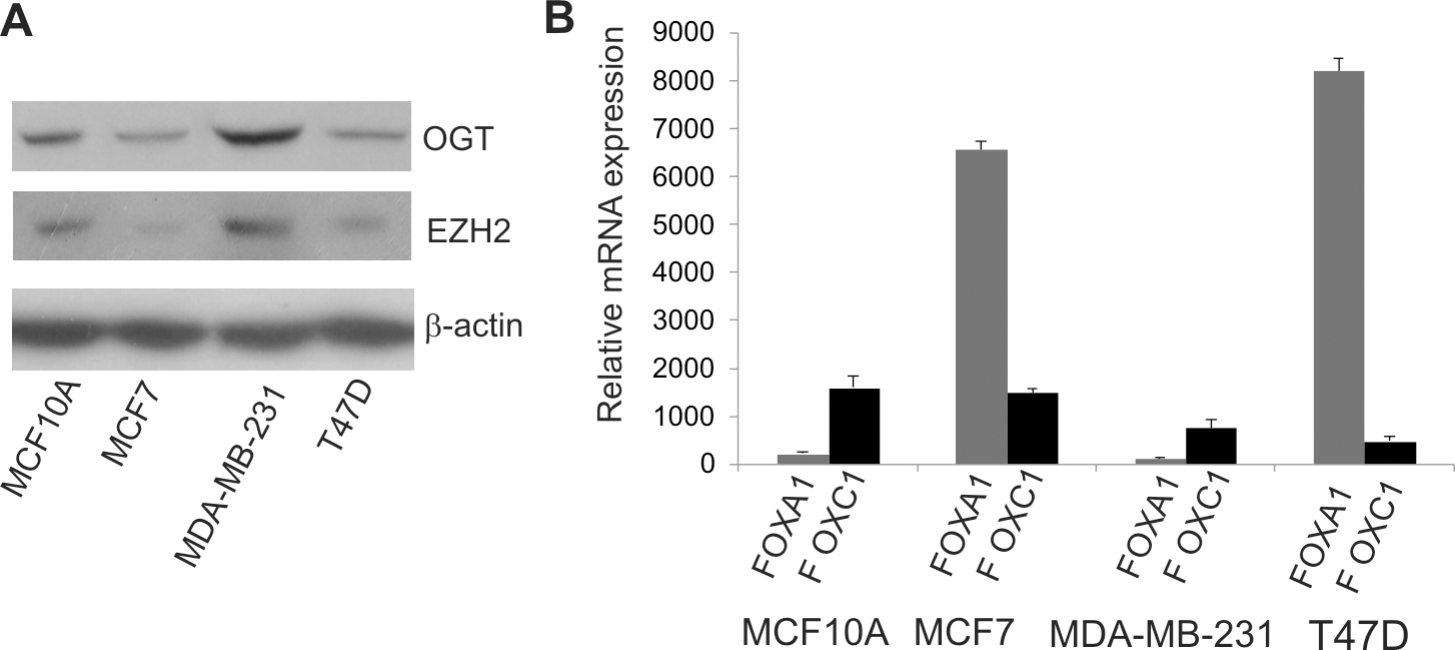
Expression of OGT, EZH2, *FOXA1* and *FOXC1* in non-malignant MCF10A cells and breast cancer cells MCF7, T47D and MDA-MB-231. A) EZH2 and OGT protein levels were analyzed by Western blot. B) The relative mRNA expression levels of *FOXA1* and *FOXC1* were determined by RT-PCR. Results are mean +/- SD from three independent experiments.

The expressions of OGT and EZH2 proteins in three cancer breast cell lines (MCF7, T47D and MDA-MB-231) and one non-neoplastic breast cell line (MCF10A) were compared (Fig 1A). As EZH2 is thought to be a critical regulator of cell migration/invasiveness higher expression of EZH2 in more aggressive cancer cells than less aggressive and non-tumorigenic cells was expected. The expression of EZH2, as expected was higher in MDA-MB-231 (more aggressive, less differentiated cells) compared to MCF7, T47 and MCF10A cells. The highest expression of OGT protein was also shown in MDA-MB-231 cells.

*FOXA1* and *FOXC1* mRNAs expression levels were compared in breast cells. *FOXA1* was highly expressed in MCF7 and T47D cells (Fig 1B). The low expression of *FOXA1* was found in MCF10A and the lowest expression of *FOXA1* was in MDA-MB-231 cells. These results are in agreement with the findings suggesting that *FOXA1* expression positively correlates with ER-positivity in breast cancers [10]. MCF7 and T47D cells fell to luminal A subtype and show high expression of ER. MDA-MB-231 cells represent claudin-low triple negative breast cancer cells and immortalized non-tumorigenic MCF10A basal breast cells do not express ER [18,19]. FOXC1 expression was higher in MCF10A and MCF7 than in T47D and MDA-MB-231.

### Effect of EZH2 and OGT down-regulation in breast cells

**Fig 2:**
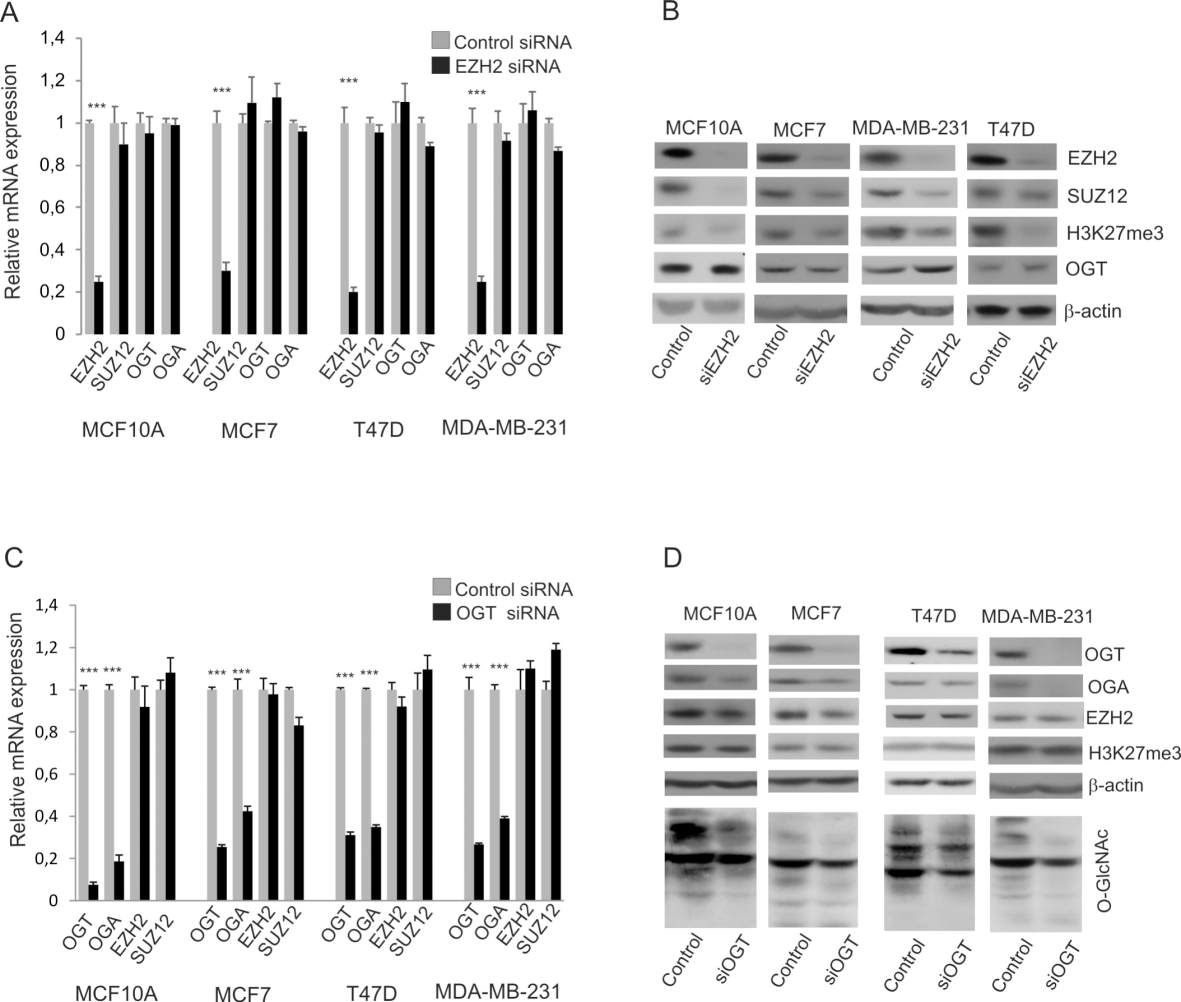
EZH2 and OGT down-regulation in breast cancer cells. The relative mRNA levels of *EZH2*, *SUZ12*, *OGT* and *OGA* were determined by real time RT-PCR in cells treated with control siRNA and EZH2 siRNA (A) or OGT siRNA (C). The bar graphs show the results as means +/-SD for experiments performed in triplicate. *** P values < 0.001. In cells with EZH2 knockdown the protein level of EZH2, SUZ12, OGT and the level of H3K27 trimethylation were determined by Western blotting (C). In cells with OGT depletion the OGT, OGA, EZH2, H3K27me3 and O-GlcNAc levels were analyzed (D).

The EZH2 or OGT were depleted in cells by siRNA. In cells treated with siRNA specific for EZH2 transcript level was substantially reduced (by 70–80%) and expression of EZH2 protein detected by Western blot was at very low level (Fig 2A and 2B, respectively). The results of densitometric analysis of bands are shown in S1 Table. The knockdown of EZH2 in all cell types caused a decrease of SUZ12 protein, which is a partner component of EZH2 in PRC2 complex without affecting its mRNA level (Fig 2A and 2B, S1 Table). This is consistent with the previous report that depletion of EZH2 may lead to destabilization of the whole PRC2 complex and decrease in level of SUZ12 [14]. As expected, decreased expression of EZH2 was correlated with decreased level of H3K27 trimethylation (Fig 2B, S1 Table). EZH2 down-regulation did not affect expression of OGT (Fig 2A and 2B, S1 Table).

**Fig 3:**
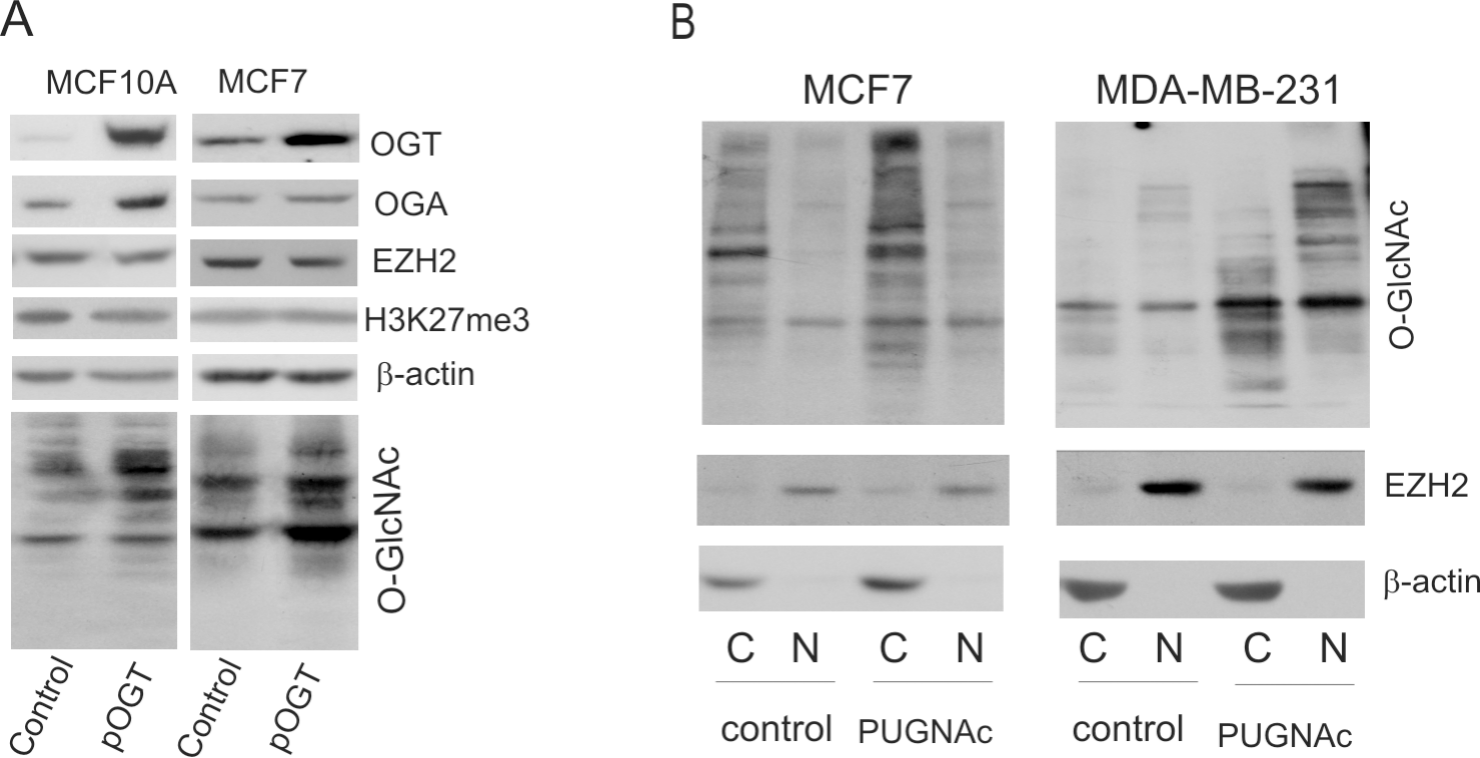
Increased O-GlcNAcylation level in breast cells caused by OGT overexpression or PUGNAC treatment does not increase EZH2 level. In order to induce overexpression of OGT, the cells were treated with plasmid vector pCMV-OGT and after 48 h the protein level of EZH2 and histone methylation level were determined by Western blotting (A). To estimate whether increased O-GlcNAcylation affects EZH2 protein expression or localization cells were treated with PUGNAc for 48 h. The O-GlcNAc and EZH2 levels in cytoplasmic and nuclear fractions were determined by Western blotting (B).

Chu et al [14] found that O-GlcNAcylation regulates EZH2 protein stability and function. They showed that EZH2 was O-GlcNAcylated and that modification stabilized EZH2 and facilitated the formation of H3K27me3 in MCF7 cells. We used siRNA to down-regulate the OGT expression. In cells treated with siOGT, the OGT transcript level was significantly reduced and expression of protein was at very low level (Fig 2C and 2D). The results of densitometric analysis of the bands are shown in S2 Table. The knockdown of OGT caused also a decrease of O-GlcNAc level in cells. The depletion of OGT was accompanied by reduced level of OGA, an OGT sister enzyme responsible for the removal of GlcNAc residues (Fig 2C and 2D, S2 Table). The knockdown of OGT caused slight decrease in protein expression of EZH2 but only in MCF7 cells the decrease of EZH2 protein level was significant (S2 Table). Despite of decreased level of EZH2 we did not observed significant decrease in histone H3K27 trimethylation (Fig 2D, S2 Table). To further clarify the impact of OGT on EZH2 we used plasmid expression vector to up regulate the OGT expression. But the cells with up regulated OGT expression did not show significant increase in EZH2 protein level (Fig 3A). We found that both down- and up-regulation of OGT expression, changed substantially the expression of OGA, which is an enzyme involved in the removal of O-GlcNAc residues (Fig 2C and 2D, Fig 3A and S1 Fig). Thus, the effect of OGT expression changes might be mitigated by changes of OGA expression. Since a weak effect of OGT deregulation on EZH2 may be due to the fact that it is accompanied with the changes in OGA level we used PUGNAc, an inhibitor of O-GlcNAcase to find out whether increase of global O-GlcNAcylation can increase the level of EZH2. The cells were additionally fractionated to determine whether the increase of global O-GlcNAcylation level can affect localization of EZH2. However, PUGNAc treatment of cells did not cause increase of EZH2 amount or affect EZH2 localization (Fig 3B). To check the effect of OGT on EZH2 distribution the control cells and cells treated with siOGT were fractionated as well (S2 Fig). The results showed mostly nuclear localization of EZH2 but slight decrease of EZH2 was observed only in cytoplasmic fraction. These results may explain why in siOGT treated cells the methylation level was not changed.

### Both EZH2 and OGT affect FOXA1 and FOXC1 expression

**Fig 4:**
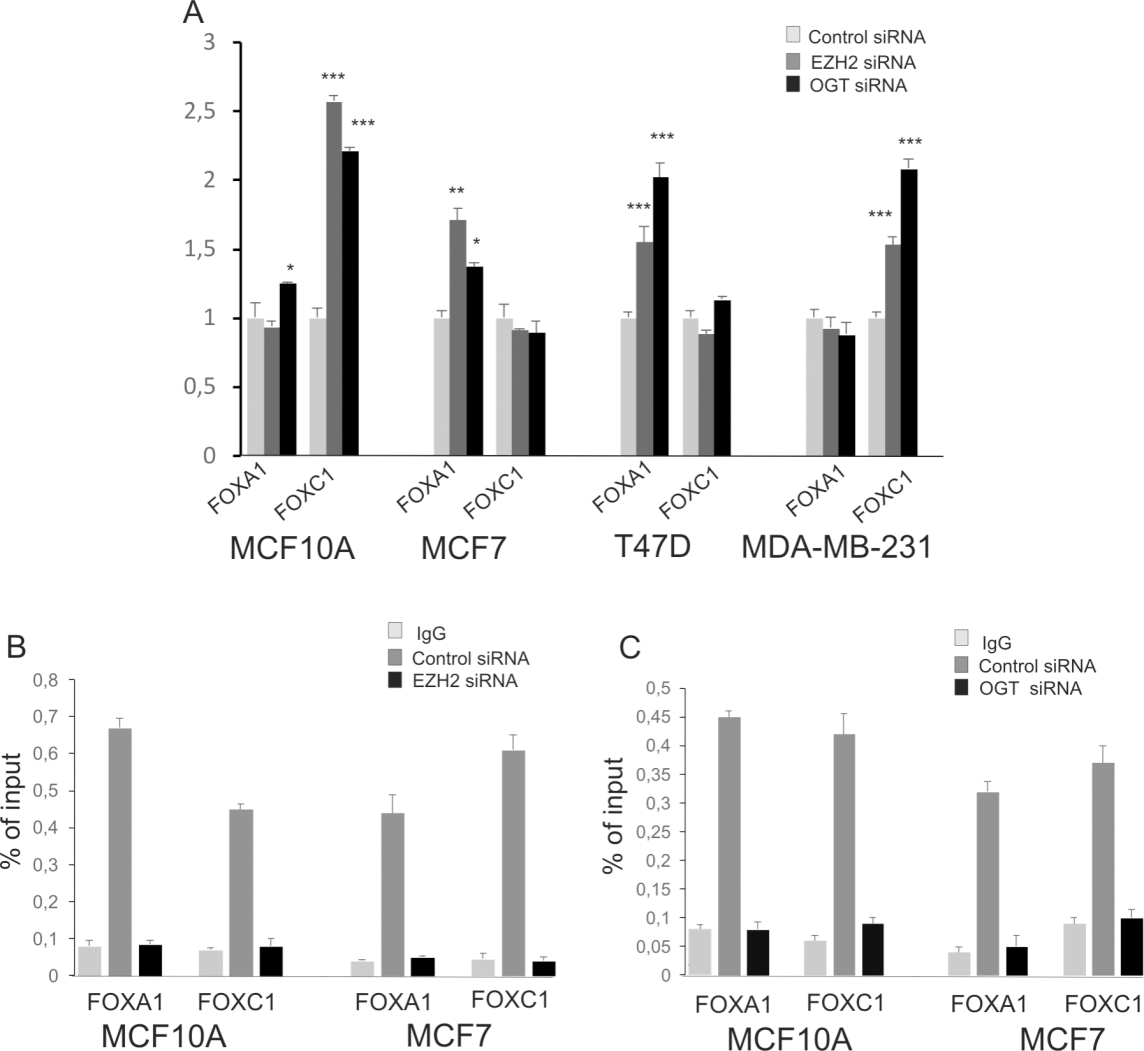
EZH2 and OGT bind to *FOXA1* and *FOXC1* promoters and affect their expressions. Cells were treated with control siRNA and siRNA specific for EZH2 or OGT and after 48h the level of *FOXA1* and *FOXC1* mRNAs were estimated by real-time RT-PCR (A). ChIP assay was performed to examine the EZH2 (B) or OGT (C) binding to *FOXA1* and *FOXC1* promoters. The figure shows the means +/- standard deviations for three experiments performed in triplicate. The asterisks indicate values of expression that were significantly different in cells with EZH2 or OGT knockdown compared to control cells; * P values of < 0.05; ** P values of < 0.01, *** P values < 0.001.

*FOXA1* was up-regulated in MCF7 and T47D cells with reduced EZH2 expression but *FOXC1* expression was not changed (Fig 4A). On the contrary, the expression of *FOXA1* did not changed and *FOXC1* was upregulated in MCF10A and MDA-MB-231 cells (Fig 4A). The effect of OGT deregulation on expression of *FOXA1* and *FOXC1* was also cell-dependent (Fig 4A). OGT depletion caused similar to EZH2 depletion effect on *FOXC1* expression in MCF10A and MDA-MB-231 cells. The OGT down-regulation caused an increase of *FOXA1* expression in MCF10A, MCF7 and T47D cells (Fig 4A).

The ChIP assay was used to check the binding of EZH2 and OGT to promoter regions of the *FOXA1* and *FOXC1* (Fig 4B and 4C). The scheme of *FOXA1*and *FOXC1* promoter regions and positioning of primers used for ChIP is shown in S3 Fig. The results showed specific binding of EZH2 to chromatin in promoter regions of *FOXA1* and *FOXC1*. In cells treated with siRNA against EZH2 the level of anti-EZH2 antibody binding level was very low compared to cells treated with scrambled siRNA (Fig 4B). ChIP assay confirmed also the binding of OGT to promoter regions of the *FOXA1* and *FOXC1* (Fig 4C).

### OGT and EZH2 affects each other’s binding to *FOXC1* promoter

**Fig 5:**
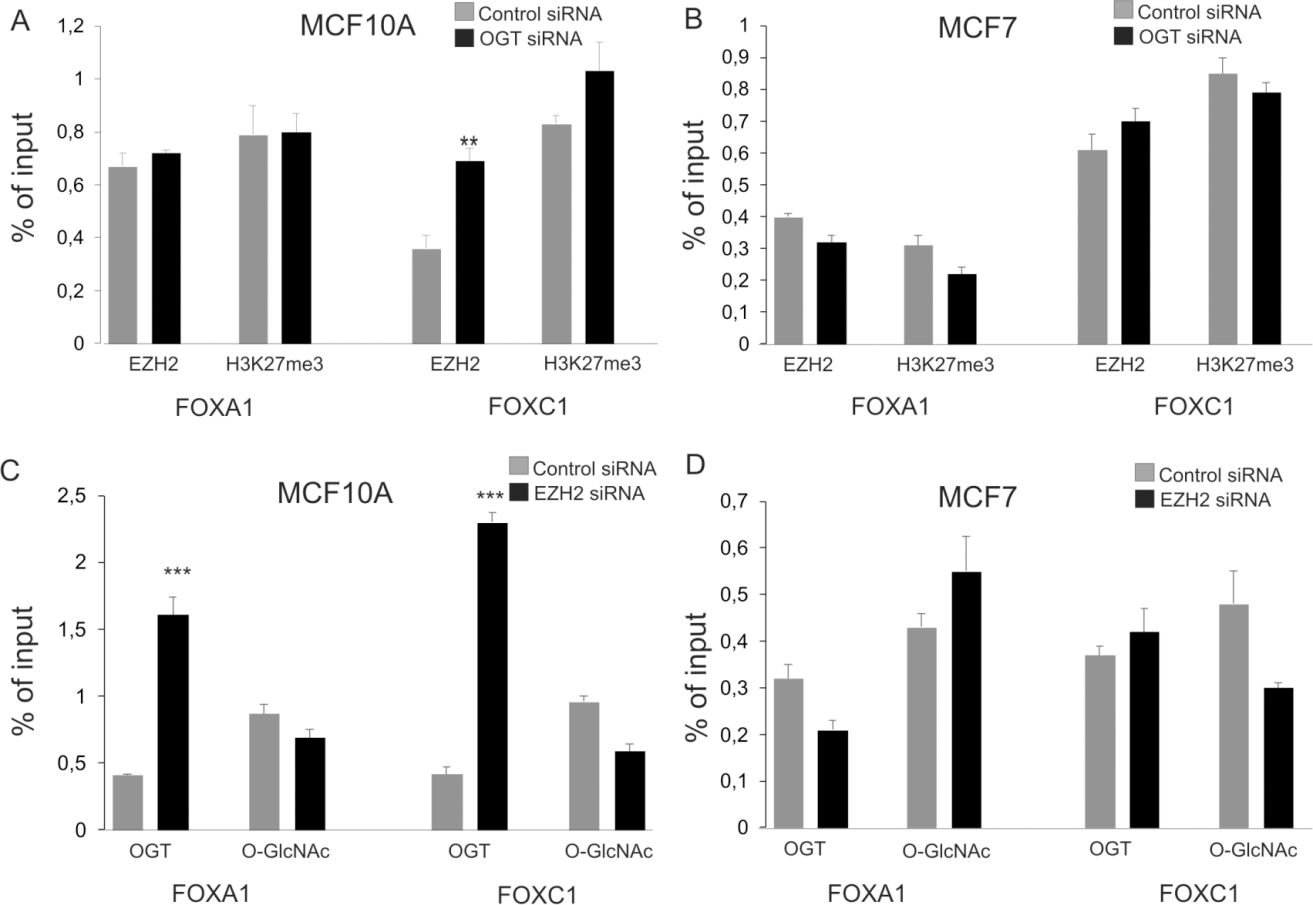
Effect of EZH2 and OGT depletion on their binding to promoter regions of *FOXA1* and *FOXC1*. ChIP assay was performed to examine the effect of OGT depletion on EZH2 binding to the *FOXA1* and *FOXC1* promoters in MCF10A (A) and MCF7 (B) cells. In OGT-depleted MCF10A and MCF7 cells histone H3 methylation level was determined (A and B). C and D–effect of EZH2 depletion on OGT binding to *FOXA1* and *FOXC1* in MCF10A (C) and MCF7 (D) cells. Data are means +/- SD of three independent assays;* P values of < 0.05; ** P values of < 0.01, *** P values < 0.001.

The chromatin immunoprecipitation assay was performed to examine whether the OGT knockdown affects EZH2 binding to *FOXA1* and *FOXC1* loci. There were no changes in EZH2 binding to *FOXA1* but the binding of EZH2 to *FOXC1* promoter region was higher in MCF10A cells treated with siOGT compared to control cells (Fig 5A). The results obtained for MDA-MB-231 cells were similar to those for MCF10A (S4 Fig). Increased binding of EZH2 in cells with depletion of OGT was not associated with significant increase in methylation of histone H3 in gene promoter (Fig 5A). We did not observe significant differences in EZH2 binding to FOXA1 and FOXC1 promoter in MCF-7 as well as T47D cells (Fig 5B and S4 Fig). Taking into account that increased binding of EZH2 to promoters in OGT-depleted cells may be due to competition between EZH2 and OGT, we checked whether EZH2 depletion affects OGT binding. We found out that OGT binding was significantly increased in MCF10A cells with knockdown of EZH2 but not in MCF7 cells (Fig 5C and 5D). However increased OGT binding did not impact significantly on O-GlcNAcylation level of chromatin proteins.

**Fig 6:**
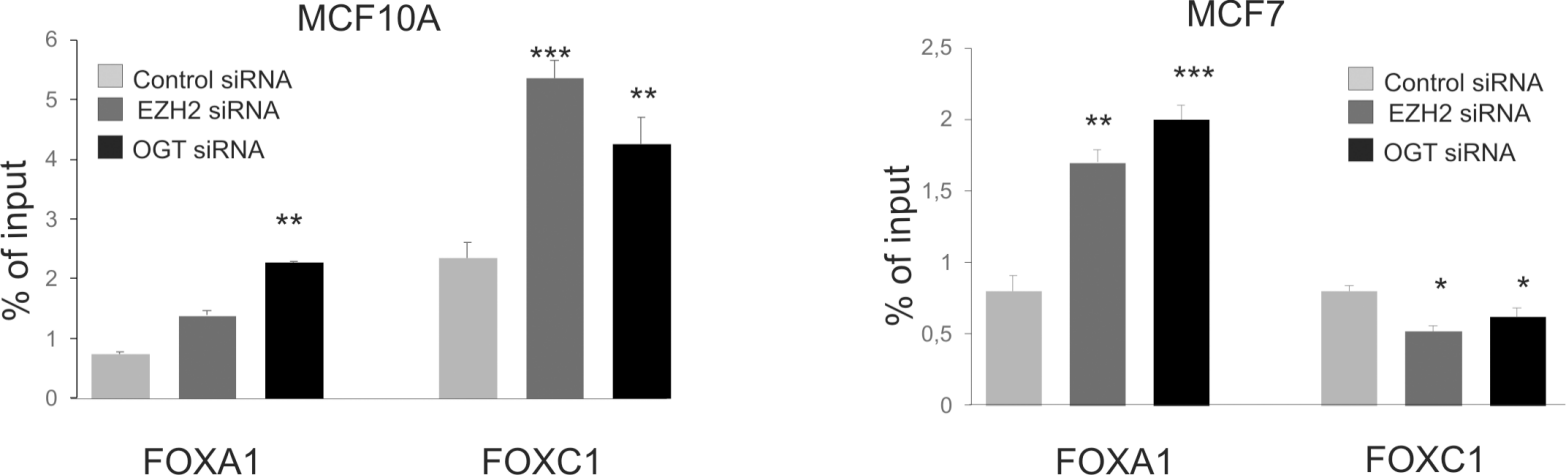
Effect of OGT or EZH2 knockdown on acetylation level of histone H3 in promoter regions of *FOXA1* and *FOXC1* genes. ChIP assay was performed in triplicate. Data are means ±SD.; * P values of < 0.05; ** P values of < 0.01, *** P values < 0.001.

As in cells treated with siOGT or siEZH2 the expressions of studied genes were increased and the methylation or O-GlcNAcylation of histones did not change significantly we check another histone modification, i.e. acetylation which is usually associated with transcription activation. It is known that both OGT and EZH2 may form complexes with other enzymes responsible for modifications of histones, especially histones deacetylases [20, 21]. Yang et al. [20] showed that OGT interacts with a histone deacetylase complex by binding to the corepressor mSin3A in HepG2 liver carcinoma cells. They suggested that OGT and mSin3A cooperatively repress transcription simultaneously with histone deacetylation. Recent studies have indicated that EZH2 interacts with HDAC1 and 2, indirectly through another PRC2 protein, EED and that activity of the HDACs is important for transcriptional repression by EZH2 (21). The anti-H3Ac antibodies were used to check acetylation changes of histone H3 in cells treated with siOGT or siEZH2. The results showed increased level of histone H3 acetylation in case of *FOXC1* gene in MCF10A. Thus, the increased expression of *FOXC1* gene was associated with increased acetylation of histone H3 in promoter region of *FOXC1*. Moreover, in MCF7 cells treated with siOGT or siEZH2 the level of histone H3 acetylation was significantly higher than in control cells (Fig 6). These results indicated that EZH2 or OGT deregulation is associated with changes of histone H3 acetylation level in in promoter regions of *FOXA1* and *FOXC1*.

### Effect of OGT deregulation on cell migration and invasiveness

**Fig 7:**
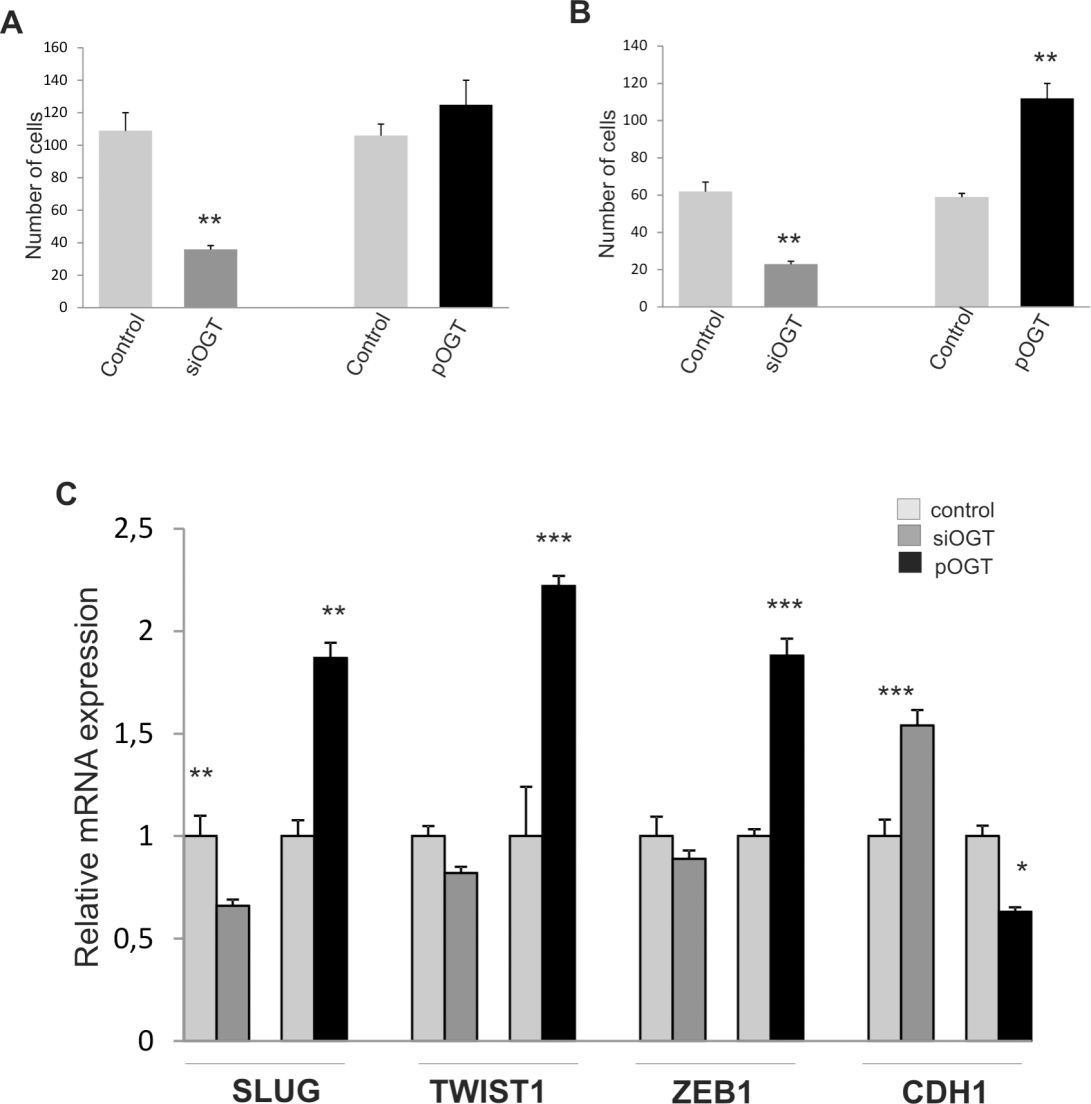
Changes in OGT expression affect cell migration and invasiveness. Migration and invasiveness assays were performed using Millicell^TM^ hanging cell culture inserts (PET membranes with 8 μm pores). Twenty-four hours after seeding of MCF10A control cells and cells transfected with siOGT or pOGT, migrated cells were visualized by Giemsa staining. For invasiveness test cell were seeded in chambers coated with Matrigel^®^ Matrix Basement Membrane. Mean values (±SD) of the migrated cells (A) or invasive cells (B) are reported. In cells with OGT knockdown or overexpression the relative mRNA expression levels of *SLUG*, *TWIST1*, *ZEB1* and *CDH1* were determined by RT-PCR (C). The figure shows the means +/- standard deviations. The asterisks indicate values of expression that were significantly different in cells with OGT knockdown or OGT overexpression compared to control cells; * P values of < 0.05; ** P values of < 0.01, *** P values < 0.001.

Increased expression of *FOXC1* was shown to be associated with changes in expression of genes involved in epithelial to mesenchymal transition (EMT) and increased cellular invasion, migration and proliferation [11–13]. In contrast to these results, Du et al [8] have found that FOXC1 inhibits metastasis of breast cancer cells. Thus, we checked whether OGT down-regulation causing increased expression of FOXC1 can affect cell migration and invasive abilities. The results showed that reduced expression of OGT in MCF10A caused decreased cell migration ability (Fig 7). Expression analysis of EMT markers showed increased expression of *CDH1* and decreased expression of *SLUG* in OGT-depleted cells (Fig 7C) The migration and invasion abilities of the OGT overexpressing cells were significantly increased (Fig 7A and 7B). *SLUG* expression was increased and *CDH1* decreased in cells with OGT overexpression. Cells overexpressing OGT showed also increased expression of *TWIST1* and *ZEB1* (Fig 7C).

## Discussion

Our results confirmed the role of EZH2 in regulation of *FOXA1* and *FOXC1* expression in breast cancer cells but the effect of EZH2 depletion on these genes expression was cell- dependent. Oncogenic function of EZH2 is mainly associated with gene silencing through the methylation of H3K27 [1]. Several reports showed that EZH2 could methylate substrates other than H3K27 and had also methyltransfrase-independent function. Recently, it was suggested that EZH2 could function as a transcription activator as well [22]. In castration resistant prostate cancer, phosphorylation of EZH2 by AKT1 at serine 21 allows EZH2 to interact with the androgen receptor at target genes and EZH2 is involved in transcription activation [23]. The role of EZH2 in repression or activation of gene expression may depend on cell type and may be context-specific. The studies of Lee et al. [24] showed that EZH2 played a role in the constitutive activation of NF-κB target genes in ER–negative basal like breast cancer cells and that function was independent of its histone methyltransferase activity. In contrast, EZH2 interacted with ER and repressed NF-κB target gene expression by inducing H3K27me3 on their promoters in ER positive luminal like breast cancer cells. Interestingly, our results showed different effect of EZH2 interference on expression of *FOXA1* and *FOXC1* in MCF7 or T47D cells, that are luminal-like ER positive cells and MDA-MB-231 cells, that are claudin-low triple negative cells. The effect of EZH2 interference in non-malignant cells MCF10A that are ER-negative was similar to MDA-MB-231 cells. The expression levels of both genes were affected in similar way by OGT interference. Chu et al. [14] found that OGT regulated EZH2 stability by O-GlcNAcylation and OGT depletion caused decreased EZH2 and histone methylation levels. Our results showed slight decrease in EZH2 level however, the changes were mainly restricted to cytoplasmic EZH2 (S3 Fig) and OGT down-regulation did not have significant impact on histone methylation level. Because changes in OGT expression entrails some changes in OGA expression the global O-GlcNAcylation level did not change much. Thus, we used PUGNAc to increase protein O-GlcNAcylation level. But PUGNAc treatment did not impact EZH2 level in breast cells. It is worth mentioning that OGT does not always affect EZH2 in mammalian cells. Myers et al. [25] showed that OGT knockdown did not affect EZH2 and H3K27me3 in mouse embryonic stem cells. Levels of PRC2 components and H3K27me3 level were found to be unperturbed in Ogt mutant *Drosophila* [26].

OGT deregulation affected *FOXA1* and *FOXC1* expressions in breast cells. The effect of OGT deregulation on EZH2 binding to promoters of these genes was cell-dependent. In MCF10A and MDA-MB-231 cells but not in MCF7, OGT depletion caused increased EZH2 binding to promoter regions. Interestingly OGT was bound to the promoter regions of *FOXA1* and *FOXC1* and EZH2 depletion increased OGT binding to these regions but only in MCF10A and MDA-MB-231 cells. These results may suggest that both EZH2 and OGT proteins are involved in regulation of *FOXA1* and *FOXC1* by interaction with other proteins and these interactions are cell-context dependent.

OGT was shown to interact with a number of transcriptional regulators. The best known role for OGT in transcriptional regulation is its involvement in Polycomb repression in *Drosophila*. Sinclair et al. [27] reported that Ogt in *Drosophila* is one of the Polycomb group (PcG) genes. Moreover, major sites on chromosomes that are modified by O-GlcNAc correspond to PcG proteins binding sites. These results suggested that OGT might play a direct role in PcG-mediated epigenetic gene silencing. Moreover, Gambetta et al. [26] found that Polycomb repression in *Drosophila* is mediated by the glycosylation of Polyhomeotic.

In addition to Polycomb OGT has been shown also to interact with mSin3A, a transcriptional repression complex associated with histone deacetylases (HDAC1, HDAC2) [18]. mSin3A and HDAC1 are O-GlcNAcylated in HepG2 liver carcinoma cells [18]. mSin3A can recruit OGT to specific genes and OGT can contribute along with HDACs to the repression of genes through the addition of O-GlcNAc modifications on transcriptional activators, inhibiting their activity. Similarly, Cox and Marsh [28] suggested that components of the mSin3A/HDAC1/2 chromatin-modifying complex can interact with OGT in harts of diabetic mice.

The results of our study showed that the acetylation level of histones in promoter regions of *FOXA1* and *FOXC1* genes was higher in breast cells with OGT knockdown than in control cells. This may suggest that OGT is involved in gene repression by regulation of histone deacetylases activity or binding.

The results of this study indicate that role of OGT in regulation of EZH2 target genes *FOXA1* and *FOXC1* is not associated with regulation of EZH2 stability. OGT itself binds to the promoter regions of studied genes and OGT knockdown may affect EZH2 binding. The two genes seem to be regulated differently and mechanism of these genes regulation in breast cancer is cell-specific. Further studies are needed to elucidate the exact role of OGT in repression of these genes.

## Supporting information

S1 Table Results of densitometric analysis of bands corresponding to EZH2, SUZ12, H3K27Me and OGT in control and siEZH2 treated cells. The bands corresponding to proteins were analyzed in Gel Pro 3.0 Analyzer software (Media Cybernetics) by measuring of integrated optical density (IOD) of the bands. We applied in lane normalization using β-actin as an internal reference. The results are presented as a mean relative IOD±standard deviation.
(DOCX)

S2 Table Results of densitometric analysis of bands corresponding to OGT, OGA, EZH2 and H3K27Me in control cells and siOGT treated cells. The bands corresponding to proteins were analyzed in Gel Pro 3.0 Analyzer software (Media Cybernetics) by measuring of integrated optical density (IOD) of the bands. We applied in lane normalization using β-actin as an internal reference. The results are presented as a mean relative IOD±standard deviation.
(DOCX)

S1 Fig The relative mRNA expression levels of OGT and OGA in cells treated with plasmid vector. Results are mean ±SD from three independent experiments.
(TIF)

S2 Fig Analysis of OGT silencing on EZH2 expression and localization. EZH2 protein level was analyzed in cytoplasmic and nuclear fractions of control cells and cells treated with siOGT for 48 h. Proteins were visualized on X‑ray film by an enhanced chemiluminescence method. Due to huge difference in EZH2 amount between nucleus and cytoplasm long and short exposure time was applied.
(TIF)

S3 Fig Schematic representation of the locations of PCR primers used for ChIP experiments. (EPS)

S4 Fig Comparison of ChIP assay results of EZH2 binding to *FOXA1* and *FOXC1* promoters in different cell lines. The figure shows the means +/- standard deviations for three experiments performed in triplicate. The asterisks indicate values of expression that were significantly different in cells with OGT knockdown compared to control cells; ** P values of < 0.01, *** P values < 0.001.
(TIF)
