# Supplementary material for: Impact of OGT deregulation on EZH2 target genes FOXA1 and FOXC1 expression in breast cancer cells
Source: PLoS One. 2018 Jun 4;13(6):e0198351. doi: 10.1371/journal.pone.0198351 (PMC5986130; doi:10.1371/journal.pone.0198351)
Supplement: S1 Table — The bands corresponding to proteins were analyzed in Gel Pro 3.0 Analyzer software (Media Cybernetics) by measuring of integrated optical density (IOD) of the bands. We applied in lane normalization using β-actin as an internal reference. The results are presented as a mean relative IOD±standard deviation. (DOCX) [file pone.0198351.s001.docx]

|  | **EZH2**  Mean ±SD p*-value* | **SUZ12**  Mean ±SD p-value | **H3K27Me**  Mean ±SD p-value | **OGT**  Mean ±SD p-value |
| --- | --- | --- | --- | --- |
| ***MCF10A***  Control  siEZH2  ***MCF7***  Control  siEZH2  ***T47D***  Control  siEZH2  ***MDA-MB-231***  Control  siEZH2 | 0.521±0.163 *0.0077*  0.049±0,021  0.563±0.118 *0.0039*  0.135±0.033  0.378±0.117 *0.012*  0.077±0.024  0.551±0.066 *0.0002*  0.020±0.027 | 0.678±0,153 *0.004*  0.036±0.01  0.208±0.075 *0.046*  0.144±0.063  0.301±0.084 *0.038*  0.127±0.039  0.232±0.023 *0.022*  0.104±0.031 | 0.178±0.07 *0.039*  0.105±0.098    0.243±0.024 *0.089*  0.186±0.016  0.837±0.230 *0.041*  0.251±0.096  0.678±0.224 *0.009*  0.080±0.046 | 0.804±0.238 *0.460*  0.622±0.159  0.393±0.114 *0.883*  0.337±0.027  0.126±0.026 *0.132*  0.093±0.015  0.193±0.010 *0.288*  0.218±0.082 |

Table S1. Results of densitometric analysis of bands corresponding to EZH2, SUZ 12, H3K27Me and OGT in control and siEZH2 treated cells

The bands corresponding to proteins were analyzed in Gel Pro 3.0 Analyzer software (Media Cybernetics) by measuring of integrated

optical density (IOD) of the bands. We applied in lane normalization using β-actin as an internal reference.

The results are presented as a relative IOD ± standard deviation
