# Supplementary material for: Impact of OGT deregulation on EZH2 target genes FOXA1 and FOXC1 expression in breast cancer cells
Source: PLoS One. 2018 Jun 4;13(6):e0198351. doi: 10.1371/journal.pone.0198351 (PMC5986130; doi:10.1371/journal.pone.0198351)
Supplement: S2 Table — The bands corresponding to proteins were analyzed in Gel Pro 3.0 Analyzer software (Media Cybernetics) by measuring of integrated optical density (IOD) of the bands. We applied in lane normalization using β-actin as an internal reference. The results are presented as a mean relative IOD±standard deviation. (DOCX) [file pone.0198351.s002.docx]

Table S2. Results of densitometric analysis of bands corresponding to OGT, OGA, EZH2 and H3K27Me in control cells and siOGT treated cells

|  | **OGT**  Mean ±SD p*-value* | **OGA**  Mean ±SD p-value | **EZH2**  Mean ±SD p-value | **H3K27Me**  Mean ±SD p-value |
| --- | --- | --- | --- | --- |
| ***MCF10A***  Control  siOGT  ***MCF7***  Control  SiOGT  ***T47D***  Control  siOGT  ***MDA-MB-231***  Control  siOGT | 0.634±0.245 0.030  0.054±0.073  0.753±0.054 0.009  0.141±0.026  0.211±0.036 0.037  0.077±0.006  0.302±0.012 <0.0001  0.029±0.014 | 0.543±0.107 0.047  0.230±0.055  0.384±0.036 0.038  0.103±0.127  0.494±0.020 0.003  0.220±0.009  0.374±0.068 0.023  0.083±0.028 | 1.003±0.145 0.064  0.745±0.287    0.342±0.048 0.023  0.196±0.011    0.392±0.161 0.840  0.298±0.127  0.705±0.135 0.106  0.582±0.030 | 0.763±0.234 0.149  0.627±0.167  0.276±0.105 0.663  0.235±0.089  0.560±0.027 0.124  0.699±0.071  0.621±0.133 0.485  0.773±0.167 |

The bands corresponding to proteins were analyzed in Gel Pro 3.0 Analyzer software (Media Cybernetics) by measuring of integrated

optical density (IOD) of the bands. We applied in lane normalization using β-actin as an internal reference.

The results are presented as a relative IOD ± standard deviation
