# Supplementary figures and images for: Impact of OGT deregulation on EZH2 target genes FOXA1 and FOXC1 expression in breast cancer cells
Source: PLoS One. 2018 Jun 4;13(6):e0198351. doi: 10.1371/journal.pone.0198351 (PMC5986130; doi:10.1371/journal.pone.0198351)

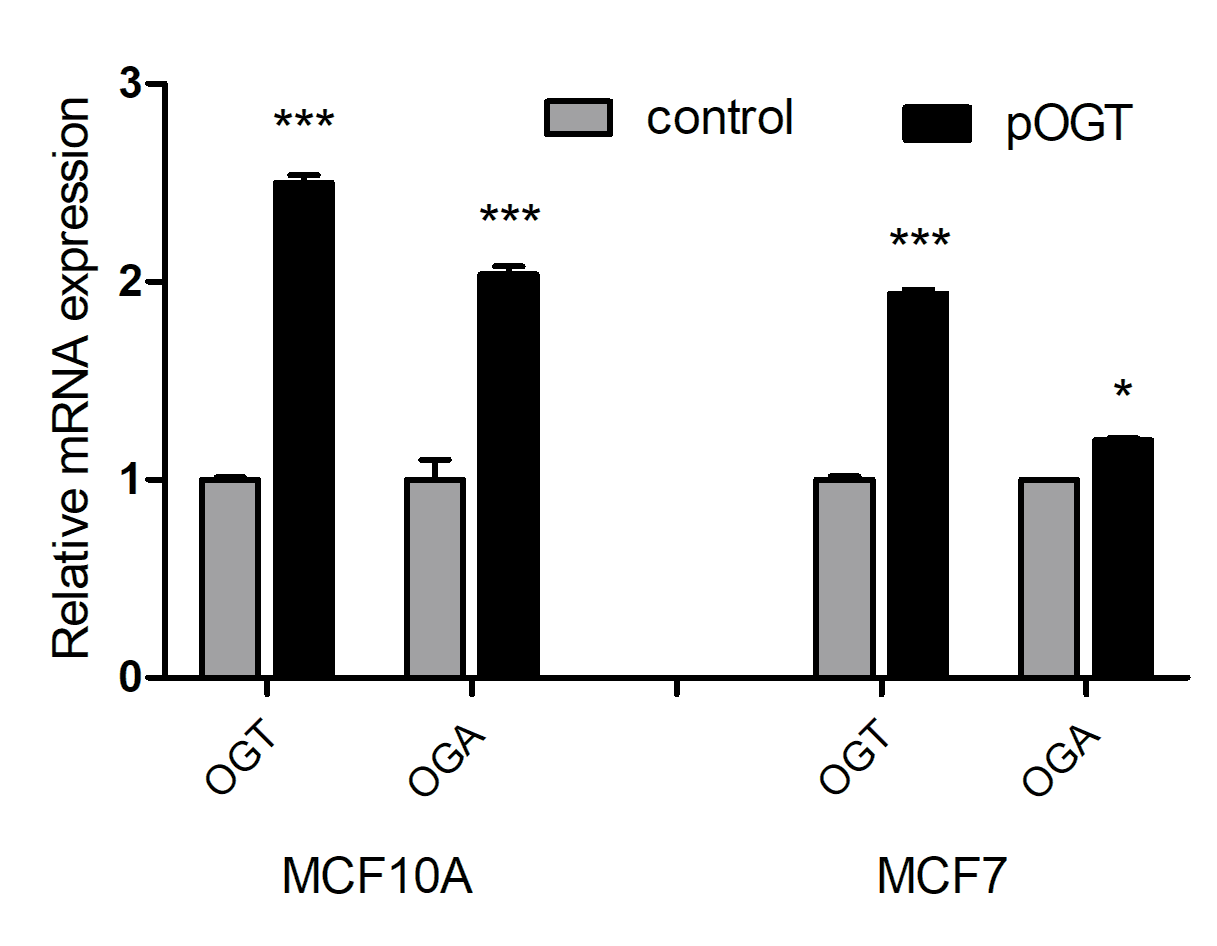

Supplement: S1 Fig — Results are mean ±SD from three independent experiments. (TIF) [file pone.0198351.s003.TIF]

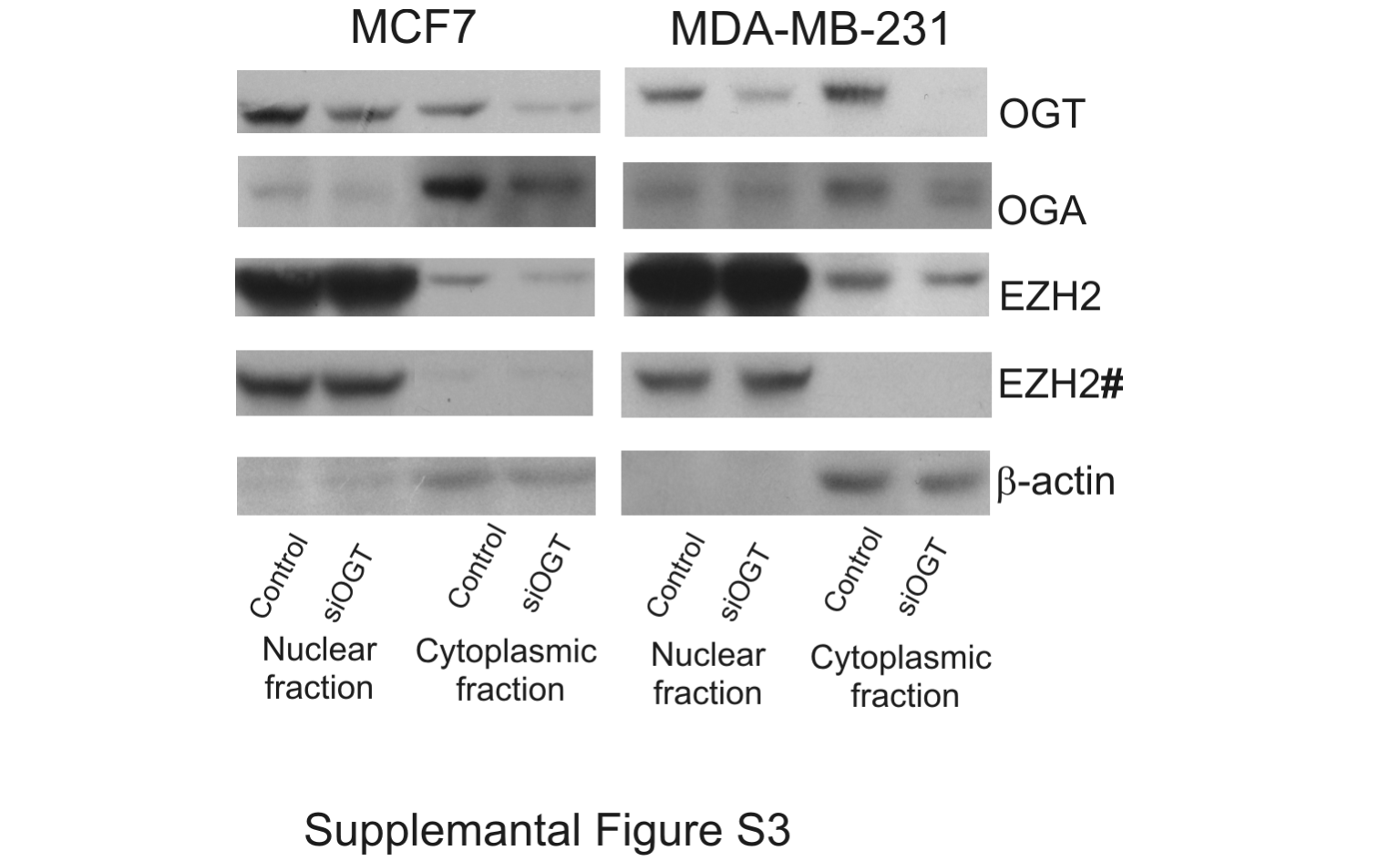

Supplement: S2 Fig — EZH2 protein level was analyzed in cytoplasmic and nuclear fractions of control cells and cells treated with siOGT for 48 h. Proteins were visualized on X‑ray film by an enhanced chemiluminescence method. Due to huge difference in EZH2 amount between nucleus and cytoplasm long and short exposure time was applied. (TIF) [file pone.0198351.s004.tif]

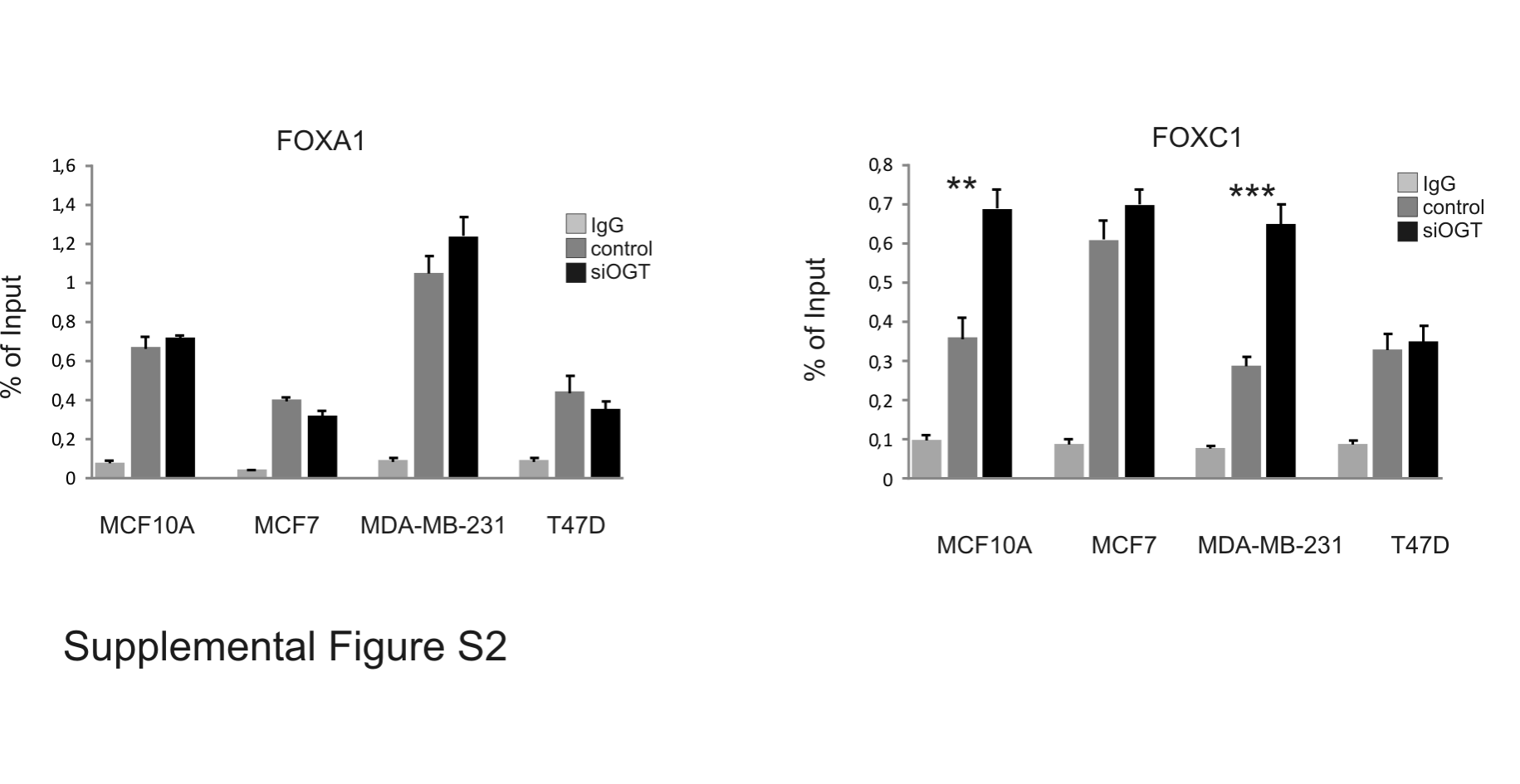

Supplement: S4 Fig — The figure shows the means +/- standard deviations for three experiments performed in triplicate. The asterisks indicate values of expression that were significantly different in cells with OGT knockdown compared to control cells; ** P values of < 0.01, *** P values < 0.001. (TIF) [file pone.0198351.s006.TIF]
